# Safety and efficacy of cell-free and concentrated ascites reinfusion therapy (CART) in refractory ascites: Post-marketing surveillance results

**DOI:** 10.1371/journal.pone.0177303

**Published:** 2017-05-16

**Authors:** Norio Hanafusa, Ayako Isoai, Tomoaki Ishihara, Tetsuya Inoue, Ken Ishitani, Taiju Utsugisawa, Toshihiko Yamaka, Tetsuya Ito, Hiroshi Sugiyama, Atsushi Arakawa, Yosuke Yamada, Yasuo Itano, Hirokazu Onodera, Ryosuke Kobayashi, Naoko Torii, Toyoko Numata, Taihei Kashiwabara, Yoshihiro Matsuno, Michio Kato

**Affiliations:** 1 Department of Blood Purification, Kidney Center, Tokyo Women’s Medical University, Tokyo, Japan; 2 Blood Purification Division, Asahi Kasei Medical Co., Ltd., Tokyo, Japan; 3 Department of Gastroenterology and Hepatology, Yokkaichi Digestive Disease Center, Mie, Japan; 4 Department of Surgery, Hita Central Hospital, Oita, Japan; 5 Department of Gynecology, Kitasato University Kitasato Institute Hospital, Tokyo, Japan; 6 Department of Transfusion Medicine and Cell Processing, Tokyo Women’s Medical University, Tokyo, Japan; 7 Department of Clinical Engineering, Faculty of Engineering, Kanagawa Institute of Technology, Kanagawa, Japan; 8 Department of Palliative Care, Japanese Red Cross Medical Center, Tokyo, Japan; 9 Department of Gastroenterology, Kizawa Memorial Hospital, Gifu, Japan; 10 Department of Obstetrics and Gynecology, Nagoya City University Graduate School of Medical Sciences, Aichi, Japan; 11 Division of Nephrology, Shinshu University Hospital, Nagano, Japan; 12 Department of Internal Medicine, Okayama Kyoritsu General Hospital, Okayama, Japan; 13 Product Vigilance and Quality Assurance Department, Regulatory Affairs, Vigilance and Quality Assurance Division, Asahi Kasei Medical Co., Ltd., Tokyo, Japan; 14 Medical Corporation, Kenseikai, Kato Michio Clinic of Liver Diseases, Hyogo, Japan; Osake University Graduate School Of Medicine, JAPAN

## Abstract

We performed post-marketing surveillance to evaluate the safety and efficacy of cell-free and concentrated ascites reinfusion therapy (CART). In total, 356 CART sessions in 147 patients at 22 centers were performed. The most common primary disease was cancer (128 cases, 300 sessions). Mean amount of ascites collected was 3.7 L, and mean concentration ratio was 9.2. Mean amount of reinfused protein was 67.8 g (recovery rate, 72.0%). Performance status, dietary intake, urine volume, body weight and abdominal circumference were significantly improved after CART. Body temperature increased significantly, by 0.3°C on average. Concomitant steroids and/or NSAIDs use before reinfusion was significantly and negatively associated with increases in body temperature. Most adverse events were fever and chills. This study examined a large number of patients compared with previous studies, and showed that CART is an effective and relatively safe treatment for refractory ascites, such as malignant ascites.

## Introduction

Cell-free and concentrated ascites reinfusion therapy (CART) is used for the treatment of ascites in patients who cannot receive higher doses of diuretics because of diuretics resistance or adverse effects. Historically, Davit et al. reported use of human ascitic fluid for transfusion in 1939 [1]. Oda et al. reported the efficacy of the prototype of CART, that is, concentrated ascites reinfusion from which cells were removed, in 1974 [2]. Since 1981 when CART was approved to be reimbursed by the National Health Insurance in Japan, CART has been used in clinical settings. CART was initially indicated for cirrhotic ascites, and has come to be widely used for malignant ascites. The reported beneficial effects of CART including amelioration of diuretic resistance, reduction of abdominal tension, and improvement in quality of life [3], and thus, it is beneficial to patients with ascites who often exhibit malnutrition. In 2001, the Kansai CART Study Group conducted a multicenter collaborative observational study to examine the safety and efficacy of CART by examining 65 sessions of CART in 25 patients with ascites, mostly due to liver cirrhosis [4]. Although many patients experienced fever during the CART procedure, which was shown to be related to the speed of ascites processing, the efficacy of CART in maintaining albumin levels was confirmed.

In the study by the Kansai CART Study Group, the majority of the patients had ascites due to cirrhosis [4]. However, CART is now widely used for patients with malignant ascites. The safety and efficacy of CART have been evaluated in several single-center studies [5–10], but only limited findings are available from a multicenter study [11]. Herein, we performed a post-marketing surveillance study to evaluate the safety and efficacy of CART mostly in patients with malignant ascites.

## Materials and methods

### Study design and ethical issues

This post-marketing surveillance by the manufacturer was conducted in accordance with the Good Post-marketing Surveillance Practice (GPSP), an ordinance of the Ministry of Health, Labour and Welfare (MHLW). GPSP is used for monitoring drugs or medical devices that have already been approved and launched in clinical settings. Obligation of establishment on documented procedures and organization system is required on marketing authorization holders when they conduct any PMS study. Therefore, the PMS study including the present one is deemed to fully compliant with ethical issues. Therefore, the approval of ethical committee for the entire protocol of this study is exempted under GPSP ordinance by MHLW. This study also conforms to the provisions of the Declaration of Helsinki (as revised in Tokyo 2004).

### Registered centers and patients

Twenty-two centers offering CART sessions were selected. To minimize selection bias, all the patients who underwent CART at each participating center between January 2014 and January 2015 were registered consecutively. Patients who underwent multiple CART sessions were registered as multiple entries. The therapeutic options for the patient, including indications for CART, were determined by the corresponding physicians in charge.

### CART procedure

The standard CART procedure comprises three steps: i) drainage of the ascites into a designated bag by abdominal paracentesis; ii) removal of malignant cells and bacteria by filtration, and removal of excess fluid and electrolytes by concentration; and iii) reinfusion of the filtered and concentrated ascites [5]. A catheter or needle was used for abdominal paracentesis to drain ascites. The AHF-MO ascitic filtration filter and AHF-UP ascitic concentration filter (both from Asahi Kasei Medical Co., Ltd., Tokyo, Japan) were used for filtration and concentration of the ascites. The direction of filtration, that is, inside-out or outside-in, and the processing conditions, such as filtration speed, concentration speed, and driving force to filter and concentrate ascites depended on individual centers.

### Survey items

Characteristics of patients examined included age, sex, primary disease (primary site in case of cancer), the presence/absence of complications diagnosed by the corresponding physicians in charge, history of ascites therapy, and concomitant use of anticancer drugs, diuretics, and steroids and/or NSAIDs (steroids/NSAIDs) before reinfusion.

The type (i.e., ascitic or pleural fluid) and the amount of collected fluid were investigated. Ascitic fluid with a serum-ascites albumin gradient (SAAG) ≥1.1 g/dL or <1.1 g/dL was regarded as transudative or exudative ascites, respectively [12]. Similarly, pleural fluid with a serum-effusion albumin gradient (SEAG) ≥1.2 g/dL or <1.2 g/dL was regarded as transudative or exudative pleural effusion, respectively [13]. Ascitic fluid and pleural effusion are hereinafter referred to as “ascites”.

The following patient characteristics were examined: bodyweight, abdominal circumference, Eastern Cooperative Oncology Group (ECOG) performance status (PS) [14], dietary intake, daily urine volume, blood test results, body temperature and blood pressure. Dietary intake was evaluated by amount of dinner intake using a 5-point scale (0%, 25%, 50%, 75% and 100%) before and after CART. Fever was defined as body temperature ≥38°C and a 1°C rise from the pre-treatment level, in accordance with the “Guide for Management of Transfusion Reactions” by the Japan Society of Transfusion Medicine and Cell Therapy [15].

Blood test included a complete blood count (white blood cells, red blood cells, hemoglobin, hematocrit, and platelets). We also investigated serum levels of total protein, albumin, total bilirubin, urea nitrogen and creatinine. It was possible that circulating blood volume could change due to the increase of colloidal osmotic pressure during the re-infusion process. Based on the assumption that the volume of the erythrocytes remained unaltered by the CART procedures, we corrected the clinical indices by the following equation taking into account the change in hematocrit (Ht) value.

Adjusted post-CART value = Post-CART value × (pre-CART Ht value)/(post-CART Ht value).

Body temperature and blood pressure were measured at four time points: before reinfusion; after completion of reinfusion; 1 h after the completion of reinfusion; a day after reinfusion.

We also investigated the driving force for filtration and concentration (pump or gravity), direction of filtration (inside-out or outside-in), duration of filtration and concentration, appearance of original ascites, and total protein and albumin levels in ascites before and after the CART procedures. The amounts of total protein and albumin in ascites were estimated by multiplying the volume of ascites by the total protein concentration and albumin concentration, respectively. The recovery rates were calculated based on the proportion of the amount of total protein after concentration compared with that of the original ascites.

All adverse events were collected that had a possible association with CART procedures, as determined by physicians in charge of the patients. We classified adverse events into two categories: those associated with ascites collection and those associated with reinfusion. Adverse events were recorded in accordance with the Medical Dictionary for Regulatory Activities / Japanese version 18.0 (MedDRA/J version 18.0), Japan Maintenance Organization, 2015.

### Statistical analysis

All data were expressed as mean ± standard deviation (SD) or proportion (%). Continuous data were analyzed by Student’s t-test or one-way analysis of variance, while categorical data were analyzed by χ^2^ test or Fisher’s exact test. Paired t-test was used for the comparisons between before and after CART in the same patient. Factors associated with the recovery rate of total protein were examined by multiple linear regression, while factors influencing fever and ascites treatment were examined by multiple logistic regression analyses. Missing data were excluded from all analysis. P values less than 0.05 (two-sided test) were considered statistically significant. Statistical analyses were performed by JMP 12.0 (SAS Institute, NC).

## Results

### Patient characteristics

**Fig 1:**
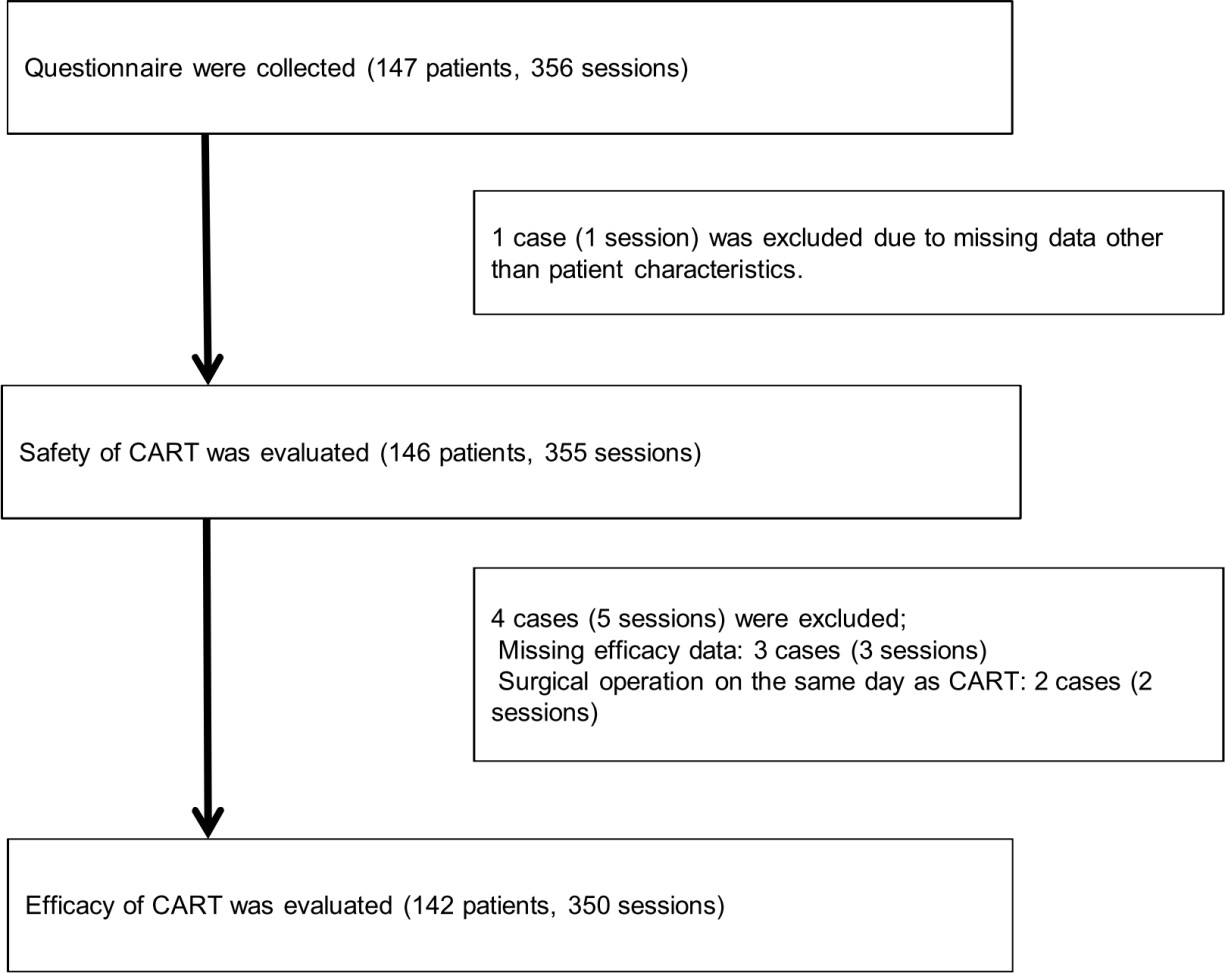
Selection of study patients. Among cases collected via a questionnaire survey, those with missing data and those who had undergone surgery on the same day as a CART session were excluded. Efficacy was examined in the remaining 142 patients (350 sessions).

Clinical data on 356 sessions of 147 patients were recovered. We excluded one patient whose data included only characteristics for safety evaluation. We further excluded five sessions with five patients for evaluation of the efficacy of CART in 350 sessions on 142 patients (Fig 1).

Characteristics of patients are shown in Table 1. Cancer was the most common primary disease (85.9%), followed by cirrhosis (11.4%). The primary sites of cancer were the ovary (25.5%), the liver and biliary tract (18.8%), the pancreas (14.8%) and the stomach (8.7%). Complications, diagnosed by the corresponding physician in charge, were found in 12.9% of cases. Renal insufficiency (10.2%) was the most common complication.

**Table 1:** Patient characteristics and CART details.

Items				Values	
Age, years				65.7±12.2 [23–90]	
Sex					
	Male			61 (41.5%)	
	Female			86 (58.5%)	
Primary Diagnoses					
	Malignancy			128 (85.9%)	
		Primary site of malignancy			
			Liver / Biliary tract		28 (18.8%)
			Pancreas		22 (14.8%)
			Stomach		13 (8.7%)
			Colon-rectum		9 (6.0%)
			Esophagus		2 (1.3%)
			Ovary		38 (25.5%)
			Uterus		5 (3.4%)
			Others		11 (7.4%)
	Liver cirrhosis			17 (11.4%)	
	Other illness			4 (2.7%)	
Complications					
	With any complications			19 (12.9%)	
		Details of complications (with redundancy)			
			Heart failure		0
			Kidney dysfunction		15 (10.2%)
			Hepatic encephalopathy		4 (2.7%)
			Esophageal varix		7 (4.8%)
			Bacterial peritonitis		0
Details of CART procedures					
	The number of sessions [session number per patient]			2.4 ± 2.7 [1–21]	
	Session interval [days]			16.6 ± 25.4 [1–246]	

CART: cell-free and concentrated ascites reinfusion therapy. Numbers in the brackets indicate the ranges.

The fields of the physicians in charge of the patients were diverse. Gastroenterology was the most common (53 patients, 36% of all patients), followed by gynecology (47 patients, 32%), abdominal surgery (23 patients, 16%), nephrology (15 patients, 10%), and medical oncology (9 patients, 6%).

The number of CART sessions per patient was 2.4 ± 2.7 during the surveillance period. Mean interval between sessions in patients who had undergone multiple CART sessions was 16.6 ± 25.4 days. Concomitant medications during CART were diuretics (178 times, 50%) and anticancer drugs (62 times, 17.4%). Steroids/NSAIDs were used before reinfusion in 157 sessions (44.5%). The objectives of CART were relief of symptoms or improvement of quality of life (274 sessions, 77.0%), reuse of autologous proteins (245 sessions, 68.8%), and continuation or resumption of anticancer therapy (46 sessions, 12.9%), with multiple answers allowed.

### CART conditions

**Fig 2:**
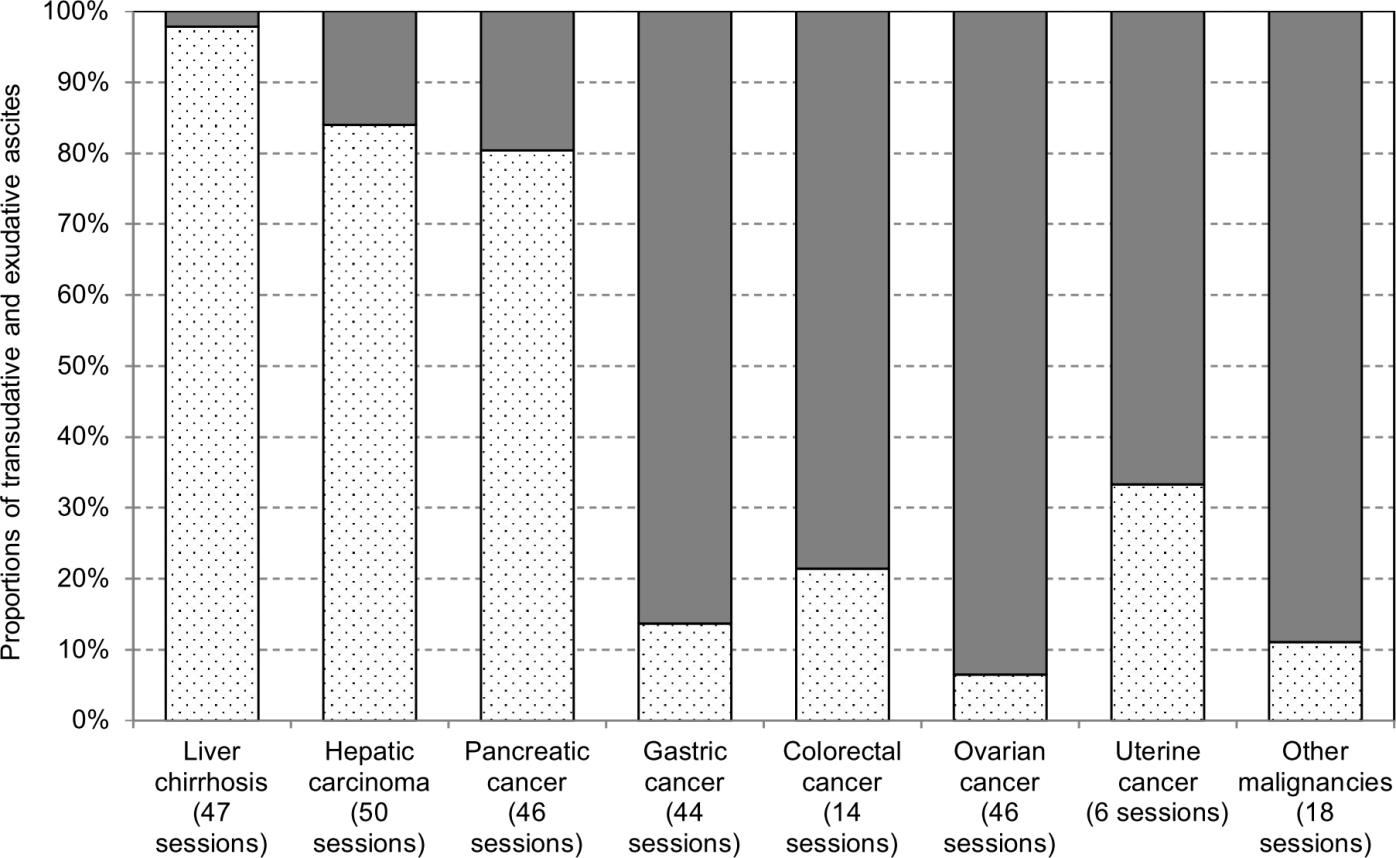
Classification by the type of ascitic and pleural fluids. SAAG or SAEG were used to classify ascitic and pleural fluids into transudative and exudative types. The transudative fluid was dominant in liver cirrhosis, liver and biliary tract cancer and pancreatic cancer, while the exudative fluid was dominant in other types of cancer. Dotted bars represent transudative fluids. Solid bars represent exudative fluid. SAAG: serum-ascites albumin gradient. SEAG: serum-effusion albumin gradient.

The conditions of 354 CART procedures are summarized in Table 2. Regarding the direction of filtration, inside-out filtration was used in 89.3% of ascitic filtration sessions. As for the driving force of CART, the pump was used in 78.2% of CART sessions. Heparin was added to the ascites collection bags in 26.6% of sessions, and the amount of heparin used was 797 ± 418 IU per 1 kg of ascites. The amount of ascites collected was 3,708 ± 1,718 g. Ascitic fluid was collected in 338 sessions, while pleural effusion was collected in 14 sessions. According to the SAAG classification, many patients with liver cirrhosis or liver and biliary tract cancer had transudative ascites, while many patients with gastric cancer, gynecological cancer or cancer of other sites had exudative ascites (Fig 2).

**Table 2:** Filtration-concentration conditions.

Items				Values	
Direction of filtration					
	Inside-out			316 (89.3%)	
	Outside-in			38 (10.7%)	
Driving force of filtration and concentration					
	Pump			277 (78.2%)	
	Gravity			77 (21.8%)	
Heparin addition into collection bags					
	Present			94 (26.6%)	
	Dosage [IU/kg ascites]				797 ± 418 [278–2,439]
Amount of collected fluid by origin					
	Overall average [g]			3,708 ± 1,718 [700–13,030]	
		Breakdowns by fluid types			
			Ascitic fluid (n = 338) [g]		3,796 ± 1,695
			Pleural fluid (n = 14) [g]		1,686 ± 891
			Mixed ascites and pleural fluid (n = 1) [g]		1,800
			Not specified (n = 1) [g]		4,100

Numbers in the brackets indicate the ranges.

### Efficacy of CART procedures

The recovery rates of ascites proteins were examined in 350 sessions of 142 patients. The characteristics of the ascites before and after CART procedures are shown in Table 3. The volume of processed ascites was 491 ± 320 g after CART: the concentration ratio was 9.2 ± 4.9 in terms of volume. The levels of total protein and albumin were 13.6 ± 6.6 g/dL and 7.4 ± 3.8 g/dL, and the calculated amounts of total protein and albumin were 67.8 ± 41.6 g and 37.8 ± 24.7 g, respectively. The recovery rates of total protein and albumin were calculated as 72.0 ± 18.1% and 73.8 ± 16.9%, respectively. After CART, the calculated amounts of total protein and albumin in transudative ascites were 49.2 ± 31.1 g and 25.8 ± 18.0 g, while those in exudative ascites were 83.9 ± 43.0 g and 47.7 ± 26.0 g, respectively. The amounts of both total protein and albumin were significantly higher in the exudative ascites than in the transudative ascites (P< 0.001).

**Table 3:** Amount and composition of original and processed ascites.

Items	Original ascites	Processed ascites
Amount of ascites (n = 348) [g]	3,709 ± 1,730 [700–13,030]	491 ± 320 [100–3,150]
Concentration ratio (n = 348)	9.2 ± 4.9 [2.0–37.0]	
Total protein concentration (n = 275) [g/dL]	2.7 ± 1.5 [0.3–8.8]	13.6 ± 6.6 [1.0–33.7]
Albumin concentration (n = 273) [g/dL]	1.4 ± 0.8 [0.1–3.7]	7.4 ± 3.8 [0.3–19.9]

Concentration ratio represents the ratio of the concentration in the original ascites to that in the processed ascites. Numbers in the brackets indicate the ranges.

**Fig 3:**
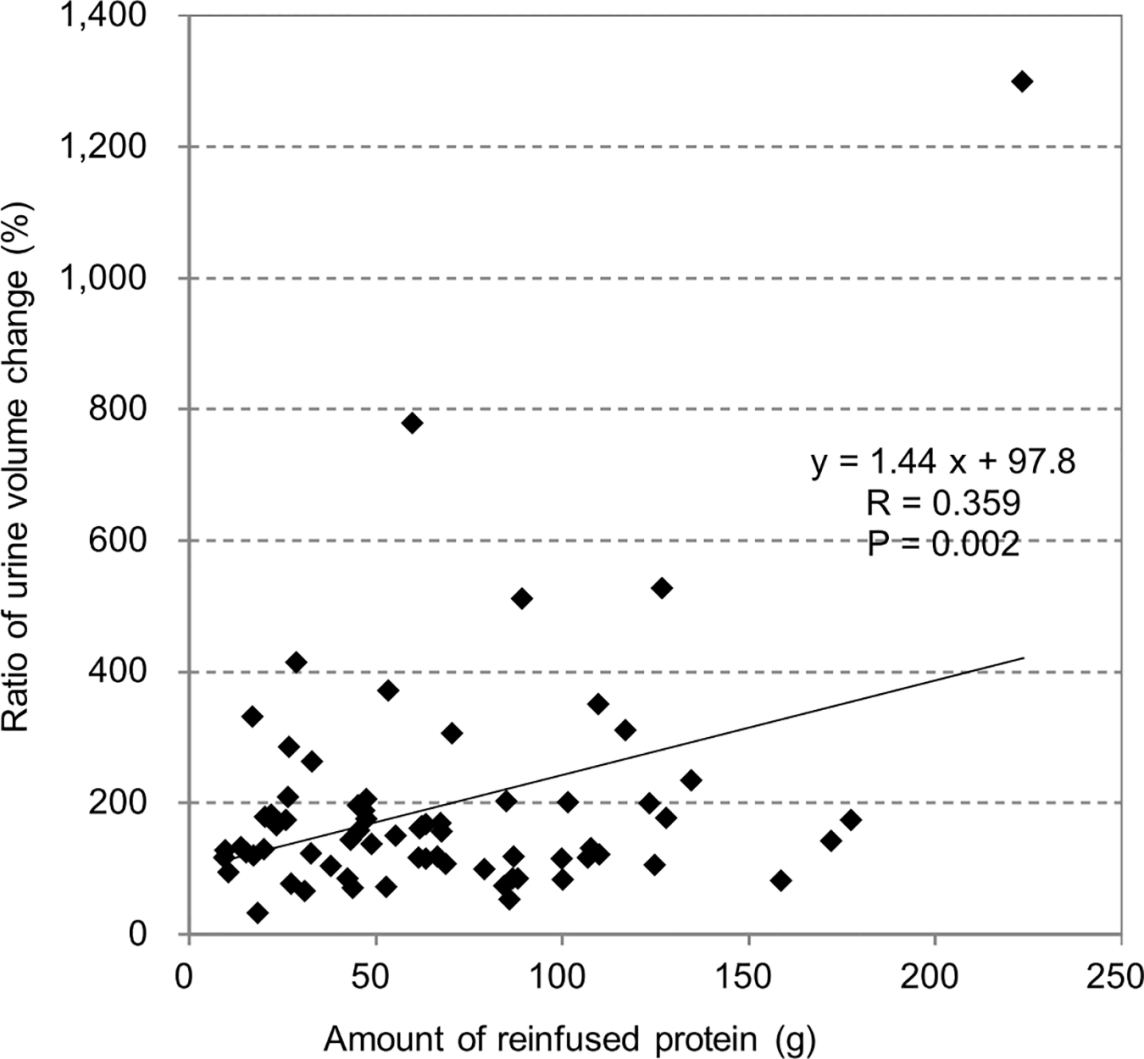
Amount of reinfused protein and change in urine volume. There was a significant positive correlation between the amount of reinfused protein and change in the urine volume.

Next, clinical changes after CART compared with status before CART are shown in Table 4. Significant positive effects were observed on all patient characteristics, namely, body weight, abdominal circumference, ECOG PS, dietary intake and daily urine volume. The changes in the urine volume between pre- and post-CART positively correlated with the amount of reinfused total protein (R = 0.359, P = 0.002), suggesting that replenishment of autologous protein may lead to increases in the urine volume (Fig 3).

**Table 4:** Changes of clinical indices between pre- and post-CART.

Items		Pre-CART	Post-CART	P-value
Patient characteristics				
	Body weight (n = 209) [kg]	56.0 ± 15.1	52.4 ± 14.6	< 0.001
	Abdominal circumference (n = 171) [cm]	89.4 ± 12.6	81.6 ± 13.3	< 0.001
	ECOG PS (n = 317)	2.2 ± 1.0	2.0 ± 1.1	< 0.001
	Dietary intake (n = 296) [%]	48.0 ± 31.5	55.6 ± 32.5	< 0.001
	Daily urine volume (n = 98) [mL]	815 ± 491	1,151 ± 666	< 0.001
Serum biochemistry corrected by Ht changes				
	Total protein (n = 227) [g/dL]	5.9 ± 0.9	6.6 ± 1.2	< 0.001
	Albumin (n = 249) [g/dL]	2.5 ± 0.5	2.9 ± 0.9	< 0.001

ECOG PS: Eastern Cooperative Oncology Group performance status.

Next, we divided the patients into tertiles by the recovery rate of total protein in their first CART session in order to investigate factors affecting the recovery rates of total protein by CART process. The ranges of recovery rates were 18.0–59.3%, 59.8–79.9% and 80.0–100% for the first, second and third tertile groups, respectively. The relationships between the total protein recovery ratios and individual factors are shown in Table 5. The first tertile group had a higher proportion of exudative ascites, higher protein levels and fibrin clot formation in the original ascites, compared with the other groups. Stepwise multiple linear regression revealed that the recovery rates were significantly related to the volume and total protein amount of the original ascites, the addition of heparin into collection bags, the direction of filtration, ascites processing speed and concentration ratios (Table 6, multiple correlation coefficient R = 0.658, coefficient of determination R^2^ = 0.433, P < 0.001).

**Table 5:** Factors influencing the recovery rate of total protein.

Items		Entire population (n = 111)	1st tertile (n = 37)	2nd tertile (n = 37)	3rd tertile (n = 37)	P-value
Recovery rate [%]		69.0 ± 19.3	47.3 ± 11.1	70.0 ± 6.0	89.8 ± 6.7	<0.001
Properties of original ascites						
	Amount of fluid [g]	3,498 ± 1,590	3,198 ± 1,659	3,436 ± 1,641	3,869 ± 1,376	0.193
	Total protein concentration [g/dL]	2.9 ± 1.6	3.6 ± 1.8	2.9 ± 1.4	2.2 ± 1.4	0.002
	Transudative ascites	45.5%	35.5%	33.3%	67.6%	0.006
	Bloody ascites	18.9%	21.6%	24.3%	10.8%	0.291
	Chylous ascites	10.1%	13.9%	8.1%	8.3%	0.727
	Fibrin clot formation	18.0%	32.4%	16.2%	5.4%	0.011
	Heparin addition in bags	32.4%	24.3%	37.8%	35.1%	0.422
Filtration-concentration condition						
	Pump use as the driving force	78.4%	81.1%	81.1%	73.0%	0.620
	Inside-out as direction of filtration	89.2%	86.5%	89.2%	91.9%	0.927
	Processing speed settings [g/min]	75.2 ± 61.4	69.0 ± 52.7	81.1 ± 71.6	75.4 ± 57.6	0.717
	Concentration ratio	8.7 ± 4.8	9.1 ± 4.8	8.2 ± 4.8	8.9 ± 4.8	0.703

Total protein concentration, transudative ascites and presence of fibrin clot formation were significantly correlated with total protein recovery rate.

**Table 6:** Factors influencing the recovery rate of total protein by multivariate analyses.

Items		β values	P-value
Properties of original ascites			
	Amount of fluid [kg]	0.273	0.002
	Total protein concentration [g/dL]	-0.591	< 0.001
	Exudative fluids by SAAG/SEAG classification	-0.157	0.199
	Chylous ascites	-0.123	0.139
	Fibrin clot formation	-0.115	0.183
	Heparin addition into collection bags	0.359	< 0.001
Filtration-concentration condition			
	Inside-out as direction of filtration	-0.410	< 0.001
	Processing speed settings [g/min]	-0.214	0.024
	Concentration ratio	-0.360	< 0.001

Multivariate analysis revealed that the protein recovery rate was associated with amount of collected ascites, total protein concentration, administration of heparin, direction of filtration.

### Safety aspects of CART procedures

#### Changes in blood pressure and body temperature

**Fig 4:**
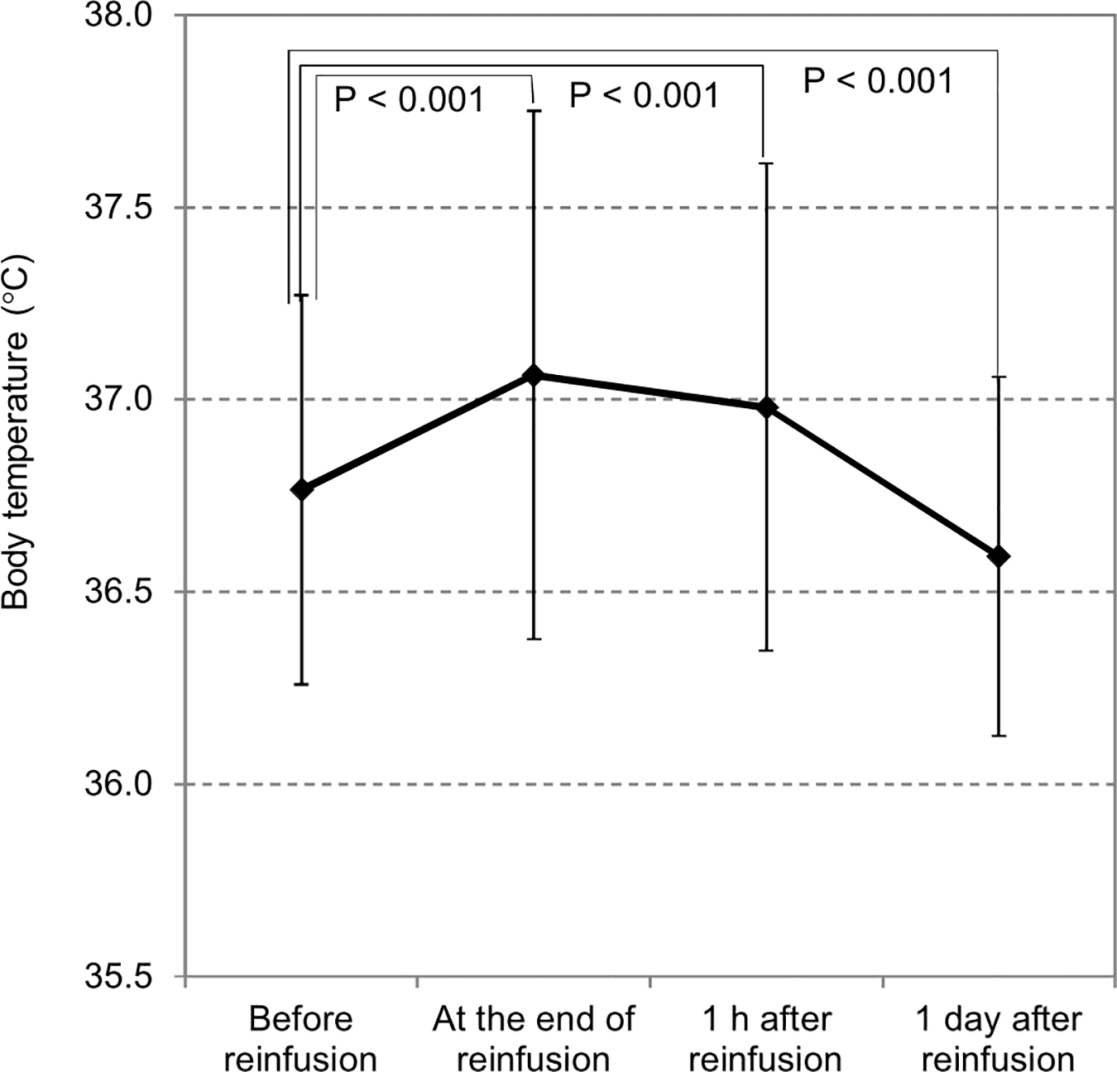
Changes in body temperature before and after reinfusion. Body temperature was significantly higher upon completion of reinfusion than before reinfusion. On the other hand, body temperature was significantly lower one day after reinfusion than before reinfusion, though neither change was clinically significant.

**Fig 5:**
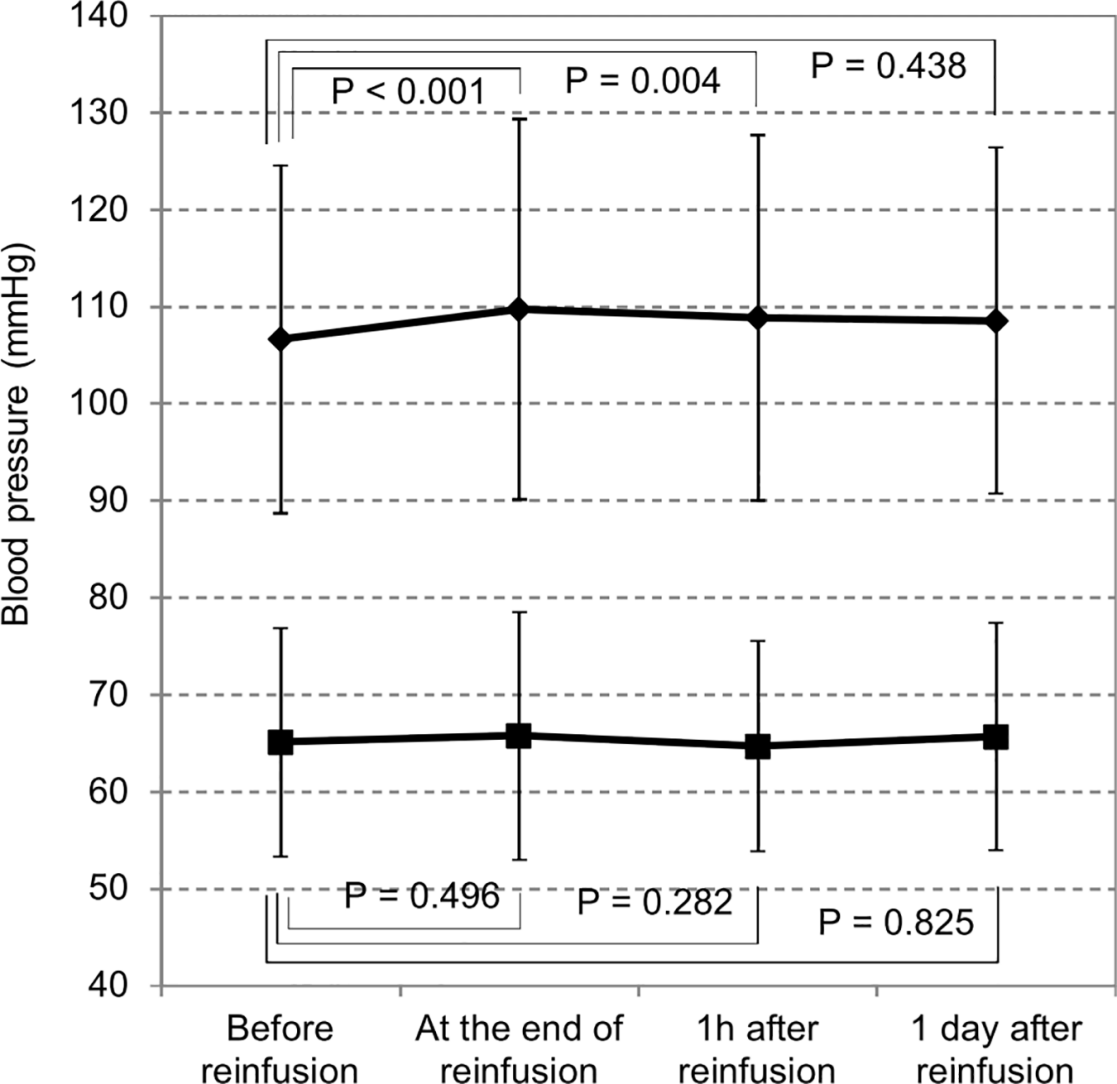
Changes in blood pressure before and after reinfusion. Systolic blood pressure was significantly higher upon completion of reinfusion than before reinfusion, while there were no significant changes in diastolic blood pressure. Diamond: systolic blood pressure. Square: diastolic blood pressure.

Changes in body temperature and blood pressure during CART are shown in Figs 4 and 5. Body temperature was elevated significantly immediately and 1 h after the completion of reinfusion, which reverted to the pre-treatment level 1 day later. Although significant changes in the systolic blood pressure were observed immediately and 1 h after the completion of reinfusion. The increase in blood pressure was small, and it suggests that reinfusion process had only minor effects on hemodynamics.

**Fig 6:**
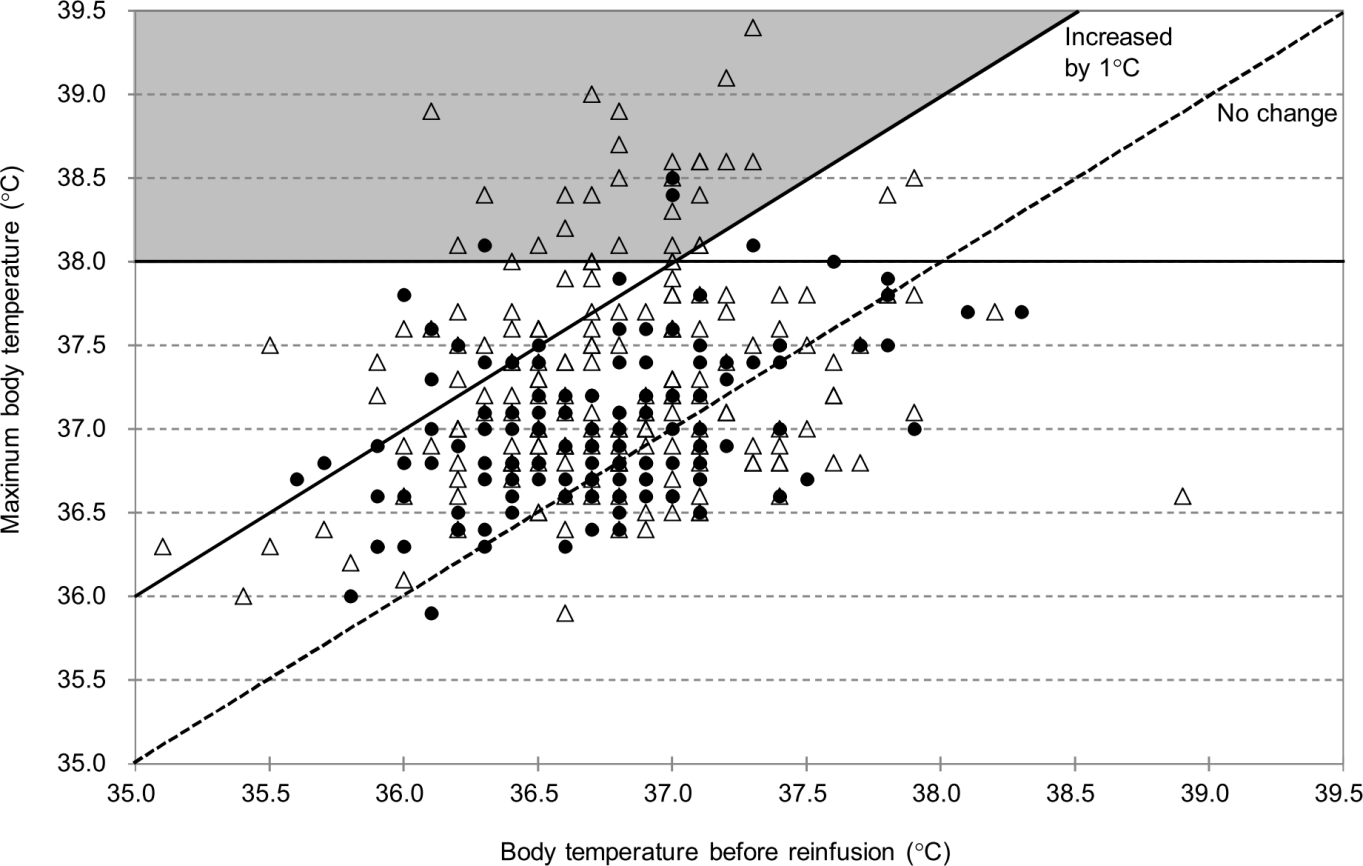
Body temperature before reinfusion and maximum body temperature: Comparison between the groups with and without use of steroids/NSAIDs. Many cases without steroids/NSAIDs were found in the shaded area indicative of defined fever (body temperature ≥ 38°C and a 1°C increase from the pre-treatment level). A solid line indicates temperature 1°C above the pre-reinfusion level, while a dotted line indicates the same temperature as the pre-reinfusion temperature. Closed circles: with concomitant use of steroids/NSAIDs. Open triangles: without concomitant use of steroids/NSAIDs.

Pre- and post-treatment body temperature data were recorded in 332 sessions and 143 patients. Significant fever was observed in only 35 CART sessions (10.5%) of 24 patients (16.8%). The relationships of significant fever with patient characteristics and reinfusion conditions were evaluated by univariate analysis (Table 7). The use of anticancer drugs, concomitant use of steroids/NSAIDs, body temperature elevation (≥37°C), white blood cell count, and the addition of heparin into collection bags were significantly different between the group with and without fever. Multivariate analysis demonstrated that concomitant use of steroids/NSAIDs before reinfusion was the only factor associated with the onset of fever (OR, 0.15; 95% CI, 0.04–0.56; P = 0.005) among the variables that were moderately associated with fever by univariate analysis (P < 0.2) (Table 8). To confirm differences between the groups with and without steroids/NSAIDs use before reinfusion, the highest temperatures after reinfusion were plotted against pre-treatment body temperatures (Fig 6). The comparison of sessions with and without steroids/NSAID indicated the proportion of patients who developed fever was significantly lower in the group with steroids/NSAIDs administration before reinfusion than in the group without (2.3% vs 19.0%, P < 0.001), and the increase in body temperature was also significantly smaller in the former group (0.2 ± 0.4°C) than in the latter group (0.5 ± 0.7°C, p<0.001). In order to investigate the differences of association with fever between NSAIDs and steroid, we further examine the incidence of fever among the patients who were given NSAIDs only and steroid only. However, the incidence of fever was so small (none among 15 patients given NSAIDs only and two among 115 patients given steroid only) and we could not determine the difference between NSAIDs and steroid (Fisher’s exact test p = 1.0).

**Table 7:** Factors associated with fever by univariate analyses.

Items		Sessions with fever (n = 35)	Sessions without fever (n = 297)	P-value
Patient characteristics				
	Elevated body temperature before CART (≥ 37°C)	51.4%	33.3%	0.034
	White blood cell counts [×10^3^/μL]	6.1 ± 3.3	7.4 ± 4.6	0.044
	eGFR<30mL/min/1.73m^2^ [%]	3.0%	10.2%	0.181
Concomitant medications				
	Diuretics	40.0%	52.5%	0.161
	Anticancer drugs	31.4%	14.5%	0.010
	Steroids/NSAIDs	8.6%	46.8%	< 0.001
Properties of original ascites				
	Transudative ascites	44.4%	52.5%	0.425
	Bloody ascites	28.6%	24.6%	0.606
	Chylous ascites	8.6%	9.6%	1.000
	Fibrin clot formation	14.3%	12.5%	0.764
	Heparin addition in bags	8.6%	26.9%	0.018
Filtration-concentration condition				
	Pump use as the driving force	85.7%	76.4%	0.214
	Inside-out as direction of filtration	91.4%	89.6%	0.731
	Processing speed [g/min]	47.9 ± 33.8	46.9 ± 27.5	0.846
	Duration of processing [min]	95.3 ± 48.9	93.8 ± 54.2	0.879
	Concentration ratio	8.6 ± 4.5	9.1 ± 4.8	0.575
Reinfusion condition				
	Amount of reinfused ascites [g]	508 ± 482	500 ± 308	0.926
	Total protein concentration for reinfusion [g/dL]	12.9 ± 4.5	13.4 ± 6.6	0.429
	Amount of reinfused protein [g]	67.2 ± 44.4	67.8 ± 41.5	0.943
	Reinfusion speed [g/hr]	111.9 ± 41.9	107.9 ± 64.4	0.629

NSAIDs: non-steroidal anti-inflammatory drugs. eGFR: estimated glomerular filtration rate.

**Table 8:** Factors associated with fever by multivariate analysis.

Items		Odds ratio (95%CI)	P- value
Concomitant medications			
	Diuretics	1.36 (0.60–3.10)	0.462
	Anticancer drugs	2.33 (0.97–5.61)	0.059
	Steroids/NSAIDs	0.15 (0.04–0.56)	0.005
Body temperature ≥ 37°C		1.84 (0.83–4.04)	0.131
White blood cell counts [×10^3^/μL]		1.00 (0.90–1.11)	0.967
eGFR value < 30mL/min/1.73m^2^		0.34 (0.04–2.80)	0.317
Heparin addition into collection bags		0.64 (0.16–2.50)	0.516

Concomitant use of steroids/NSAIDs was independently and only associated with fever. CI: confidence interval. NSAIDs: non-steroidal anti-inflammatory drugs.

**Fig 7:**
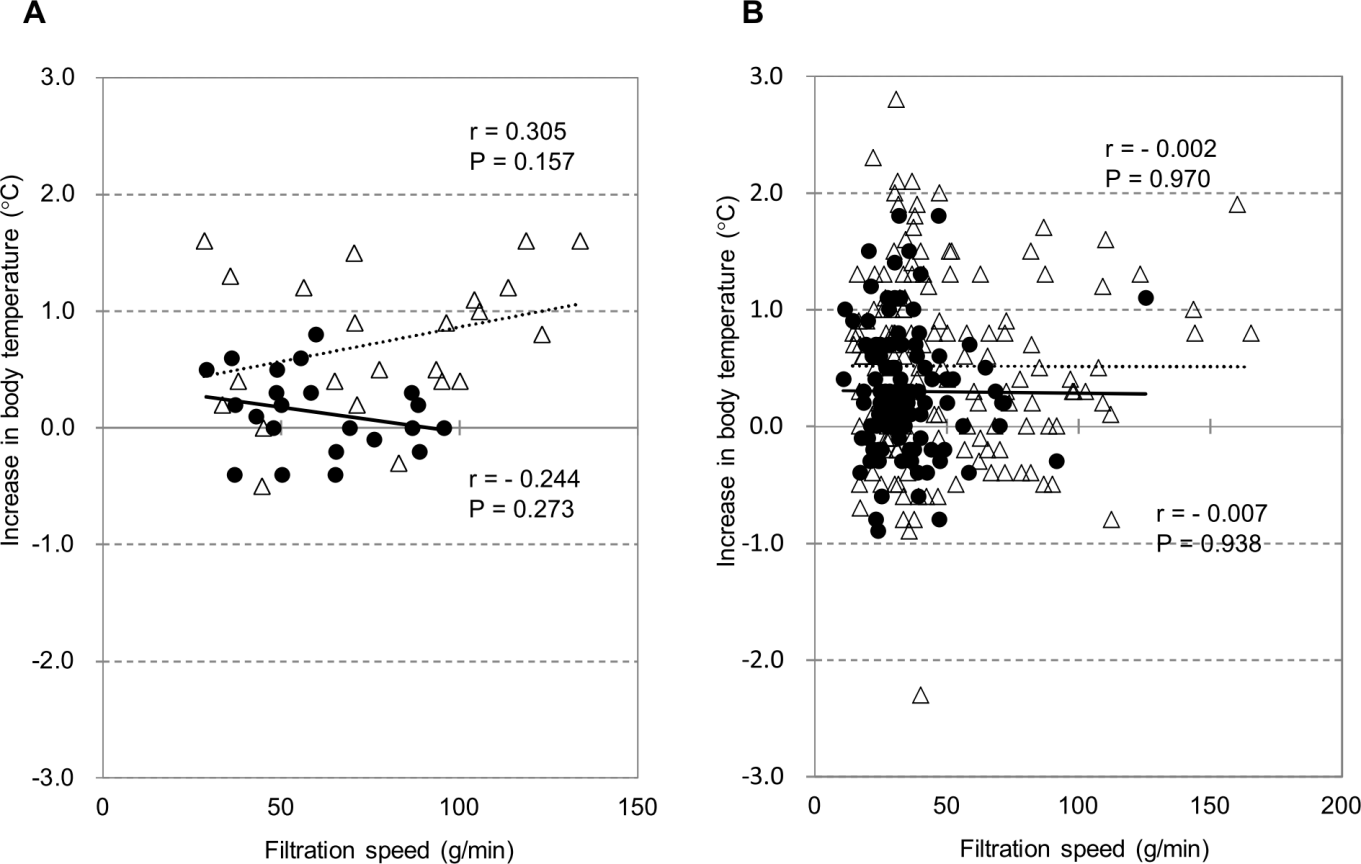
Relationship between the filtration-concentration speed and the increase in body temperature. The increase in body temperature tended to be high when the filtration-concentration speed was high only among the patients with cirrhotic ascites and without concomitant use of steroids/NSAIDs (A). However, no relationship between them was found in malignant ascites regardless of concomitant use of steroids/NSAIDs (B). Circle: with concomitant steroids/NSAIDs. Open triangles: without concomitant steroids/NSAIDs. NSAIDs: non-steroidal anti-inflammatory drugs.

The Kansai CART Study Group previously demonstrated a significant relationship between the increase in body temperature and the speed of ascites processing [4]. Although the increases in body temperature in this study population exhibited a weak positive relationship with processing speed (R = 0.305, P = 0.157) in patients with liver cirrhosis who did not receive concomitant steroids/NSAIDs before reinfusion, no correlation was observed in cancer patients irrespective of concomitant steroids/NSAIDs (Fig 7).

#### Changes in blood test values between pre- and post-CART

The blood test results of both pre- and post-CART procedures are shown in Table 9. The Ht value after CART was significantly lower than that before CART, suggesting an increase in the circulating plasma volume due to the dilution effect caused by an increase in plasma colloid osmotic pressure. Thus, individual plasma levels were corrected by the change in Ht. Total protein (6.6 ± 1.2 g/dL) and albumin (2.9 ± 0.9 g/dL) after CART were significantly higher than the corresponding pre-CART values (5.9 ± 0.9 g/dL and 2.5 ± 0.5 g/dL, respectively), indicating the effect of reinfusion of autologous proteins (Table 4). Significant changes were also observed in white blood cell count, platelet count and creatinine level, although none of them were considered clinically significant. Changes in urea nitrogen and creatinine levels were compared before and after CART procedures among patients with renal impairment. No significant difference was found in either the urea nitrogen level (pre-CART 49.9 ± 26.3 mg/dL vs post-CART 50.3 ± 25.2 mg/dL, P = 0.864) or the creatine level (pre-CART 2.9 ± 1.5 mg/dL vs post-CART 2.7 ± 1.8 mg/dL, P = 0.391) in 27 CART procedures among the patients with an estimated glomerular filtration rate (eGFR) < 30 mL/min/1.73 m^2^.

**Table 9:** Changes in blood test results between pre-and post-CART.

Items		Pre-CART	Post-CART	P-value
Complete blood cell counts				
	White blood cells (n = 306) [×10^3^/μL]	7.2 ± 4.5	8.0 ± 5.3	< 0.001
	Red blood cells (n = 306) [×10^4^/μL]	349.3 ± 62.1	348.5 ± 62.3	0.240
	Platelets (n = 306) [×10^4^/μL]	23.7 ± 14.2	22.8 ± 13.8	0.005
	Hemoglobin (n = 306) [g/dL]	10.6 ± 1.8	10.7 ± 1.8	0.172
	Hematocrit (n = 306) [%]	32.3 ± 5.2	30.1 ± 4.8	< 0.001
Serum biochemistry				
	Total bilirubin (n = 268) [mg/dL]	1.3 ± 1.9	1.3 ± 1.8	0.073
	Urea nitrogen (n = 297) [mg/dL]	25.6 ± 15.4	25.2 ± 16.6	0.319
	Creatinine (n = 299) [mg/dL]	1.0 ± 0.8	0.9 ± 0.8	0.026
	eGFR (n = 299) [ml/min/1.73m^2^]	66.6 ± 27.9	71.3 ± 30.5	<0.001

Test results, except hematocrit value itself, were corrected by the changes of hematocrit value. eGFR, estimated glomerular filtration rate.

#### Frequencies of adverse events and technical problems

Adverse events, which were evaluated by the doctors in charge, related to drainage of the ascites or reinfusion of processed ascites were separately analyzed.

Among 355 sessions of 145 patients with a complete safety dataset, adverse events related to the drainage of ascites occurred in 6.2% of patients (9/145) and 2.5% of sessions (9/355). Adverse events occurred on 9 occasions. Among the adverse events related to drainage of ascites, only one was considered severe. Other adverse events included hypotension (4 cases), chest pain (1 case), abdominal pain (1 case) and dyspnea (1 case) during/after drainage of the ascites, and hyperammonemia after reinfusion (1 case) (Table 10).

**Table 10:** Adverse events associated with CART procedures.

Adverse events	Patients	Sessions	Time point (sessions)			Severity (sessions)	
			During ascites drainage	During reinfusion	After reinfusion	Severe	Not severe
Shock	1 (0.7%)	1 (0.3%)	1	0	0	1	0
Hypotension	4 (2.7%)	4 (1.1%)	4	0	0	0	4
Chest pain	1 (0.7%)	1 (0.3%)	1	0	0	0	1
Abdominal pain	1 (0.7%)	1 (0.3%)	1	0	0	0	1
Dyspnea	1 (0.7%)	1 (0.3%)	1	0	0	0	1
Hyperammonemia	1 (0.7%)	1 (0.3%)	0	0	1	0	1
Total	9 (6.2%)	9 (2.5%)	8	0	1	1	8

Most of the adverse events associated with CART procedures were not severe, except one case of hemorrhagic shock at ascites drainage.

The severe adverse event (hemorrhage following abdominal paracentesis) occurred in a 62-year-old man with cancer of unknown primary site. Hemostasis by manual compression and blood transfusion resulted in a full recovery from the event. Adverse events were absent after reinfusion of the filtrated and concentrated ascites in this patient.

Adverse events related to reinfusion occurred in 22.6% of patients (33/146) and 13.2% of sessions (47/355): the total numbers of adverse events were 56 (Table 11). The majority of adverse events were fever and chills, but none of them was considered severe and patients fully recovered from the events.

**Table 11:** Adverse events associated with reinfusion of filtered and concentrated ascites.

Adverse events (with redundancy)	Patients	Sessions	Time point (sessions)		Severity (sessions)	
			During reinfusion	After reinfusion	Severe	Not severe
Fever	30 (20.5%)	44 (12.4%)	41	3	0	44
Chills	8 (5.5%)	8 (2.3%)	7	1	0	8
Shivering with chills	1 (0.7%)	1 (0.3%)	1	0	0	1
Nausea	1 (0.7%)	1 (0.3%)	1	0	0	1
Hypertension	1 (0.7%)	1 (0.3%)	1	0	0	1
Headache	1 (0.7%)	1 (0.3%)	0	1	0	1
Any adverse events	33 (22.6%)	47 (13.2%)	51	5	0	56

A considerable number of fever/chills cases were observed, but none of them was severe.

Technical problems with the filtration and concentration devices during operation occurred in 9.6% of patients (14/146) and 5.4% of sessions (19/355): technical problems with devices were found in 22 cases in total. The most common type of problem was a circuit pressure rise, and the sessions were prematurely terminated in 4 cases. In remaining 18 cases, the CART procedures were continued, 15 of which required some alteration of the conditions, such as reduction of processing speed or concentration ratio.

The processing was completed in 91% of CART sessions (the completed group) and terminated in 9% (the terminated group). The volume of the unprocessed portion of the ascites was 632.7 ± 536.2 g (17% of the original volume) in the termination group. Total protein level, the proportion of transudative ascites, fibrin clot formation and the addition of heparin into collection bags, processing speed setting and concentration ratio were significantly different between the completed group and the terminated group (Table 12). We further performed multivariate analysis on factors with P < 0.2 in univariate analysis. As shown in Table 13, only the total protein concentration in the original ascites was significantly associated with premature termination of CART (OR = 6.94, 95% CI 3.23–14.90, P < 0.001).

**Table 12:** Factors associated with premature termination of CART sessions (univariate analysis).

Items		Completed sessions (n = 321)	Terminated sessions (n = 33)	P-value
Properties of original ascites				
	Amount of fluid [g]	3694 ± 1741	3841 ± 1469	0.641
	Total protein concentration [g/dL]	2.4 ± 1.4	4.5 ± 1.0	< 0.001
	Transudative ascites	56.0%	15.4%	< 0.001
	Bloody ascites	25.9%	27.3%	0.860
	Chylous ascites	9.1%	9.7%	1.000
	Fibrin clot formation	10.3%	30.3%	< 0.001
	Heparin addition in bags	28.3%	9.1%	0.021
Filtration-concentration condition				
	Pump use as the driving force	79.4%	66.7%	0.090
	Inside-out as direction of filtration	88.8%	93.9%	0.555
	Processing speed settings [g/min]	87.3 ± 62.9	57.8 ± 58.7	0.010
	Concentration ratio	9.5 ± 4.9	5.3 ± 2.2	< 0.001

**Table 13:** Factors associated with premature termination of CART sessions (multivariate analysis).

Items		Odds ratio (95%CI)	P-value
Properties of original ascites			
	Total protein concentration [g/dL]	6.94 (3.23–14.90)	< 0.001
	Exudative fluid by SAAG/SEAG classification	0.20 (0.04–1.08)	0.061
	Fibrin clot formation	2.26 (0.63–8.07)	0.208
	Heparin addition in bags	0.27 (0.06–1.28)	0.098
Filtration-concentration condition			
	Pump use as the driving force	1.51 (0.32–7.01)	0.602
	Processing speed settings [g/min]	1.00 (0.99–1.01)	0.745
	Concentration ratio	0.90 (0.70–1.16)	0.415

Multiple logistic regression analysis indicated that total protein concentration was significantly associated with premature termination of CART sessions. SAAG: serum-ascites albumin gradient. SEAG: serum-effusion albumin gradient.

## Discussion

In this study, we evaluated the safety and efficacy of CART in a post-marketing surveillance setting. The majority (85.9%) of patients had malignant ascites, and CART improved all tested clinical indices (body weight, abdominal circumference, ECOG PS, dietary intake, and urine volume), presumably due to favorable protein replenishment effect. On the other hand, increases in body temperature were observed, but they were slight and transient. Preventive medication suppressed increases in body temperature. There were no clinically significant changes in blood pressure.

### Patient characteristics

Compared with several previous epidemiological surveys [4, 5, 11], this study is distinguishable as it examined a larger number of patients including a large proportion of malignant ascites patients. On the other hand, the patients in the previous multicenter study reported by the Kansai CART Study Group in 2003 were mostly cirrhotic patients [4], and thus, the primary illness is markedly different from that in this study. Our study indicated that CART is now widely used for patients with malignant ascites. Compared with cirrhotic patients, cancer patients more likely to have exudative and/or hemorrhagic ascites. SAAG is an index to differentiate exudative ascites from transudative ascites [12]. Exudative ascites was often seen in patients with gastroenterological or gynecologic cancer, while transudative ascites was seen in cirrhosis, liver and biliary tract cancer and pancreatic cancer. Liver and biliary tract cancer has a background of liver cirrhosis in many cases, and our results indicate that ascites complicated by pancreatic cancer also exhibited transudative elements. The number of CART sessions per patient was 2.4 ± 2.7 during the study period. Some patients could receive CART therapy either before or after this surveillance period and so the number of sessions could be underestimated. Nevertheless, this study revealed that most of the patients underwent multiple CART sessions. Maeda et al. reported that total protein levels were high and prognosis was favorable in the patients who underwent ≥ 5sessions [6]. Also, long intervals between CART sessions and a good prognosis were reported in gynecologic cancer patients who had undergone chemotherapy [7]. This study did not analyze prognosis, and thus, a large-scale study examining the relationship between CART and prognosis is anticipated. Mean interval between CART sessions was 16.6 ± 25.4 days in this study, probably reflecting the restriction of claims to once biweekly under the coverage of the Japanese Health Insurance.

### Efficacy

Mean concentration ratio was approximately 9.2 ± 4.9 in this study, while it was 10.2 ± 2.8 in the study examining mainly cirrhotic ascites by the Kansai CART Study Group [4]. We showed that a large proportion of cirrhotic patients had transudative ascites (Fig 2), and cirrhotic and transudative ascites was concentrated in a higher ratio (data not shown). Although this study mainly examined patients with malignant ascites, the mean concentration ratio was approximately 10 and was similar to that in the study by the Kansai CART Study Group [4]. Meanwhile, the amount of total protein recovered was 67.8 ± 41.6 g, which was similar to the level (63.12 ± 39.4 g) in a study on the patients with malignant ascites by Ito et al. [5]. Data on total protein recovery are not available in the study by the Kansai CART Study Group. The CART study group reported that the recovery rates of globulin and albumin by the outside-in devices were 71.1 ± 9.6% and 57.6 ± 7.1%, respectively [11]. The recovery rates of globulin and albumin in the present study were 72.0 ± 18.1% and 73.8 ± 16.9%, respectively, which were equivalent to or greater than the above-mentioned levels. Multivariate analysis revealed that the recovery rate is associated with the amount of ascites, total protein concentration, addition of heparin into collection bags, direction of filtration, processing speed setting and concentration ratio. The amount of ascites and use of heparin were associated with high recovery rates, while a high level of total protein, filtration using an outside-in device, fast processing speed and a high concentration ratio were associated with low recovery rates. The large amount of ascites was often transudative with low total protein content (data not shown), and this study showed that protein recovery rates were high for such ascites. A previous report showed that the recovery rate decreased in higher speed processing of the ascites [16], as is indicated by this study that the recovery rate negatively correlated with processing speed setting and concentration ratio. Taken together, it might be advisable to set the maximal speed of processing in order to increase the recovery rate during CART procedures. On the other hand, there were center-specific protocols on the direction of filtration (data not shown). Such center-specific practice patterns may have influenced the relationship between the direction of filtration and the recovery rate.

Regarding use of an anticoagulant, heparin was added in 26.6% of sessions. A practice pattern in which 500 U/L of heparin is added to the ascites was reported [5]. The averaged dosage of heparin was 797 IU/kg in this study, which is larger than in the previous study. However, a higher amount of heparin showed a tendency toward a lower incidence of premature termination of CART procedures. Heparin is expected to suppress the formation of fibrin clots in collection bags, and it is noteworthy that our univariate analysis showed a relationship of fibrin clot formation with recovery rate and premature termination of CART sessions. Nevertheless, this is an observational study and future interventional studies are needed to examine the effect of heparin administration and the direction of filtration.

The study by the Kansai CART Study Group showed no changes in albumin values [4]. On the other hand, increases in plasma osmotic pressure due to colloidal components, such as albumin, cause influx of extravascular fluid into vascular spaces [17, 18]. Such fluid movement dilutes the blood [19], leading to underestimation of the effect of CART on albumin and protein replenishment. The results corrected by the changes of Ht revealed significant increases in both total protein and albumin levels after CART, suggesting that CART actually replenished these factors in addition to the fluid influx caused by the increase of plasma colloid osmotic pressure.

The urine volume was associated with the amount of protein reinfused, and this may be explained by the fluid influx from the outside of the blood vessel. This influx increases renal perfusion and recovered renal impairment due to prerenal factors. Meanwhile, the concept of renal congestion was proposed [20], and increases in abdominal pressure are known to reduce renal function [21, 22]. Although this study did not measure abdominal pressure, a significant decrease in the abdominal tension suggested decreases in abdominal pressure, as seen in a previous study [3]. Thus, increases in the urine volume by CART may also be attributed to decreases in abdominal pressure, as well as to increases in colloid osmotic pressure.

This study also showed improvement in dietary intake and PS. CART reduces abdominal distention [3], and mechanically, this may lead to an increase in dietary intake. Alleviation of symptoms and recovery of activities in daily life were reported in a previous study [3], and this may be associated with improvement in PS. In any case, patients with a large amount of ascites often have malnutrition and low PS, and thus, increases in dietary intake and improvement of PS will be beneficial in improvement of the nutritional status, as well as in relieving symptoms.

It was previously shown that the platelet count drops during CART, and this study also showed statistically significant decreases in the platelet count even after Ht correction. However, the platelet count of 22.8 ± 13.8 × 10^4^/μL after CART was not considered clinically significant. Further, no hemorrhagic complication after CART, probably associated with decreases in the platelet number, was observed.

### Safety

Generally, fever is a clinically significant problem in CART [23], and the Kansai CART Study Group indicated that the processing speed was related to body temperature elevation [4]. Other possible factors involved are cytokines. Only mild elevation of body temperature was observed in this study, in good agreement with previous findings on patients with malignant ascites [5]. The fact that high fever was rarely observed in this study may be due to the different patient population from that in the Kansai CART Study Group (malignant vs cirrhotic ascites). Nevertheless, we performed multivariate analysis in order to identify factors associated with body temperature elevation, but we did not find that SAAG was a significant factor. On the other hand, a weak relationship of body temperature elevation with processing speed was shown in patients with cirrhotic ascites who did not take steroids/NSAIDs. In fact, steroids were used in 5 patients (12 sessions) and NSAIDs in 9 cases (23 sessions) in the Kansai CART Study Group [4]. It may be important to process ascites at a reasonable speed and to administer preventive medication when we perform CART on cirrhotic patients.

The preventive effect of steroids/NSAIDs against blood temperature elevation was shown in this study, which is consistent with findings of a previous single-center study [5]. The underlying mechanism may be the suppression of prostaglandin and inflammatory cytokines by these drugs [24]. Also, inflammatory cytokines in the ascites and blood were shown to be associated with CART-related fever [25]. Although cytokine levels were not measured, this study indicated that fever is controllable by suppressing the final pathway leading to the onset of fever with use of steroids or NSAIDs.

It was previously reported that blood pressure moderately drops upon ascites drainage, but recovers on reinfusion [5]. Data on blood pressure before drainage were not available, thus it was impossible to evaluate this effect of drainage in this study. However, pre-reinfusion blood pressure values were 106.6 ± 17.9 mmHg / 65.1 ± 11.8 mmHg, which did not indicate marked reduction. Therefore, clinically significant hypotension associated with the drainage of the ascites is unlikely. On the other hand, blood pressure tended to rise upon reinfusion, supporting previous findings. These findings suggested the possibility of urine volume increases via prerenal elements due to an increase in blood pressure.

#### Adverse events and technical problems

Adverse events and technical problems were separated by time point: during ascites drainage, reinfusion, and the filtration-concentration process. Adverse events at ascites drainage included one case of severe hemorrhagic shock following abdominal paracentesis. Although hemostasis and blood transfusion led to a full recovery from the event in this case, it is important to keep in mind that CART has a risk of bleeding caused by paracentesis, an invasive procedure. On the other hand, we found decreases in blood pressure in only four cases (approximately 1%), which was consistent with the minor changes in blood pressure described above.

The most common adverse events during reinfusion were fever and chills. Indeed, elevation of body temperature has been a matter of concerns, but clinically significant fever was relatively rare. The incidence and severity of adverse events were low during CART procedures. Taken together, these results suggest that CART is a relatively safe procedure.

Technical problems included increases in circuit pressure and clogging. These events can be linked to early termination of CART, though only 9% of cases were terminated prematurely. Moreover, the volume of unprocessed ascites was relatively small (632.7 ± 536.2 mL).

The total protein level was associated with early termination. Malignant ascites is often bloody and contains a high concentration of protein. Thus, appropriate drainage of the clogged fluid within the hollow fiber of the filtering membrane, as well as suitable monitoring, appears important in processing of malignant ascites.

#### Limitations

There are several limitations in this study. First, this is an observational study, and thus, the causal relationship of each factor with the outcome cannot be inferred. Prospective interventional studies are required in the future. Second, several indices, such as dietary intake and PS, were evaluated subjectively by medical staff at each facility, which suggests the possibility of differences of evaluation across facilities. We included an explanation of each index in the questionnaire in order to standardize evaluation, but indices that are more standardized should be considered in the future. Third, not all CART-related data of individual patients were collected. Furthermore, the possibility of selection bias cannot be eliminated, because we did not examine all CART-performing centers in Japan, although active centers were selected. Lastly, this study was conducted in a post-marketing surveillance setting, and more detailed data, such as cytokine measurements, were not available including economical aspects. Currently, the medical cost for CART procedure is 41,600 JPY for technical fee and 64,100 JPY for the devices. These costs might be more expensive than albumin infusion, depending on the amount of protein re-infused. However, CART has advantages over albumin infusion; it can obviate the needs of albumin, one of the plasma derivative products, and it can lead to the appropriate use of these products. Moreover, the components that are replenished during CART procedures other than albumin, such as globulin or other unknown factors, might have some beneficial effects on the disease process of the patients with malignancies. These points require further analyses.

Despite these limitations, this is the largest ever multicenter study examining CART-related data in a large number of patients, the status of current CART implementation and its outcome.

## Conclusion

This study evaluated the safety and efficacy of CART in the post-marketing surveillance setting. The majority of cases were malignant ascites, probably reflecting the current situation surrounding CART procedures. Implementation of CART was shown to be safe and effective even in this patient group with primarily malignant ascites. Prospective studies based on our findings are necessary to examine more detailed CART conditions in future.
